# Supplementary material for: Paternal effects without paternity? Testing non-genetic male influence on offspring size and brood size in a gynogenetic vertebrate, the Amazon molly (Poecilia formosa)
Source: PLoS One. 2026 Feb 27;21(2):e0328962. doi: 10.1371/journal.pone.0328962 (PMC12948072; doi:10.1371/journal.pone.0328962)
Supplement: S1 File — (PDF) [file pone.0328962.s001.pdf]

Supplementary Information 1: Model summaries & supporting figures

For:

Paternal effects without paternity? Testing non-genetic male influence on offspring size and brood size in a gynogenetic vertebrate, the Amazon molly (*Poecilia formosa*)

Ulrike Scherer[1,2,3]*, Sean M. Ehlman[1,2,3,4], David Bierbach[1,2,3], Jens Krause[1,2,3] & Max Wolf[1,3]

[1] SCIoI Excellence Cluster, Technische Universität Berlin, Berlin, Germany

[2] Faculty of Life Sciences, Humboldt University, Berlin, Germany

[3] Department of Fish Biology, Fisheries, and Aquaculture, Leibniz Institute of Freshwater Ecology and Inland Fisheries, Berlin, Germany

[4] Department of Biological Sciences, University of South Carolina, Columbia, SC, USA

*Corresponding author: u.k.scherer@gmail.com

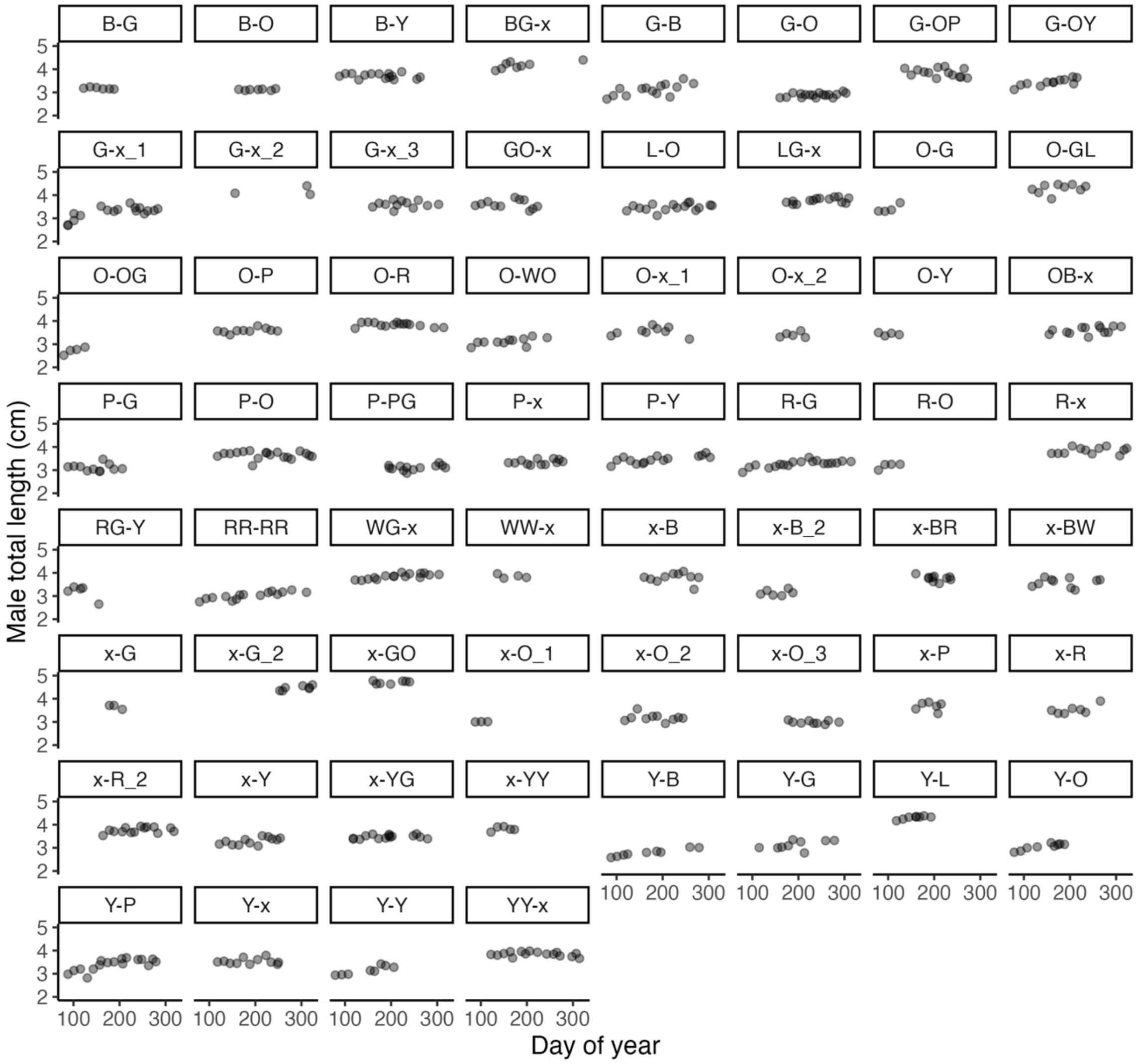

S1 Figure: Overview male size measurements. Body size of individual males (*N* = 60) was measured as their total length (cm) every time they were transferred among breeding tanks. Each panel represents a single male, with points showing individual measurements.

S1 Table: Link between primary and secondary male size and offspring size. Model summary of a linear mixed-effect model testing whether male size predicts offspring size, while controlling for female prior experimental conditions (treatment, experimental block), female size at parturition, female origin, and tank specifics (tank system, tank level, and tank centrality).

Response	*Predictors*	*Estimate*	*SE*	χ^2	*p*	*df*
Offspring size (mm)	(Intercept)	0.395	0.055	-	-	-
	Primary male body size	0.022	0.009	6.392	**0.011**	1
	Secondary male body size	0.019	0.009	4.476	**0.034**	1
	Female prior treatment [Predator]	-0.003	0.010	0.097	0.756	1
	Block [2]	-0.010	0.012	0.751	0.687	2
	Block [3]	-0.009	0.012			
	Female body size at parturition (cm)	0.045	0.008	25.029	**<0.001**	1
	Tank system [2]	0.065	0.011	26.873	**<0.001**	1
	Tank level [Level4]	-0.007	0.015	4.288	0.232	3
	Tank level [Level2]	0.007	0.013			
	Tank level [Level1]	0.023	0.013			
	Tank centrality [Periphery]	0.021	0.012	2.881	0.091	1
	**Random Effects**					
	σ^2	0.00				
	τ_{00} (Brood ID)	0.00				
	τ_{00} (Secondary male ID)	0.00				
	τ_{00} (Female/Tank ID)	0.00				
	τ_{00} (Primary male ID)	0.00				
	τ_{00} (Female origin)	0.00				
	N (Female/Tank ID)	53				
	N (Primary male ID)	50				
	N (Secondary male ID)	56				
	N (Brood ID)	127				
	N (Female origin)	6				
	Observations	2435				
	Marginal R^2 / Conditional R^2	0.393 / NA				

S2 Table: No link between male size and brood size. Model summary of a linear mixed-effect model testing whether male size predicts brood size, while controlling for female prior experimental conditions (treatment, experimental block), female size at parturition, female origin, and tank specifics (tank system, tank level, and tank centrality).

Predictors	***Predictors***	***Estimate***	***SE***	χ^2	***p***	***df***
Brood size	(Intercept)	53.509	13.038	-	-	-
	Primary male body size	-1.430	1.977	0.512	0.475	1
	Secondary male body size	-3.216	2.071	2.366	0.124	1
	Female prior treatment [Predator]	0.529	1.696	0.097	0.755	1
	Block [2]	-0.796	2.156	0.883	0.643	2
	Block [3]	-1.901	2.015			
	Female body size at parturition (cm)	-2.617	1.971	1.742	0.187	1
	Tank system [2]	2.489	1.873	1.592	0.207	1
	Tank level [Level4]	-3.066	2.683	9.102	**0.028**	3
	Tank level [Level2]	-3.375	2.216			
	Tank level [Level1]	-7.189	2.194			
	Tank centrality [Periphery]	-0.550	2.015	0.073	0.787	1
	**Random Effects**					
	σ^2	72.22				
	τ_{00} (Secondary male ID)	0.00				
	τ_{00} (Female/Tank ID)	3.99				
	τ_{00} (Primary male ID)	1.04				
	τ_{00} (Female origin)	0.00				
	N (Female/Tank ID)	53				
	N (Primary male ID)	50				
	N (Secondary male ID)	56				
	N (Female origin)	6				
	Observations	126				
	Marginal R^2 / Conditional R^2	0.152 / NA				
