# Supplementary material for: Paternal effects without paternity? Testing non-genetic male influence on offspring size and brood size in a gynogenetic vertebrate, the Amazon molly (Poecilia formosa)
Source: PLoS One. 2026 Feb 27;21(2):e0328962. doi: 10.1371/journal.pone.0328962 (PMC12948072; doi:10.1371/journal.pone.0328962)
Supplement: S2 File — (PDF) [file pone.0328962.s002.pdf]

Supporting Information 2: Robustness analysis with respect to the inclusion of Phase 1 reproduction

For:

Paternal effects without paternity? Testing non-genetic male influence on offspring size and brood size in a gynogenetic vertebrate, the Amazon molly (*Poecilia formosa*)

Ulrike Scherer[1,2,3]*, Sean M. Ehlman[1,2,3,4], David Bierbach[1,2,3], Jens Krause[1,2,3] & Max Wolf[1,3]

[1] SCIoI Excellence Cluster, Technische Universität Berlin, Berlin, Germany

[2] Faculty of Life Sciences, Humboldt University, Berlin, Germany

[3] Department of Fish Biology, Fisheries, and Aquaculture, Leibniz Institute of Freshwater Ecology and Inland Fisheries, Berlin, Germany

[4] Department of Biological Sciences, University of South Carolina, Columbia, SC, USA

*Corresponding author: u.k.scherer@gmail.com

S3 Table: No link between male size and offspring size – when including Phase 1 reproduction. The model structure is identical to the model presented in S1 Table, with the difference that offspring from Phase 1 broods were included, where no male overlap during the female's fertile window occurred (i.e., 30 ± 2 days prior to parturition).

Response	***Predictors***	***Estimate***	***SE***	χ^2	***p***	***df***
Offspring size (mm)	(Intercept)	0.519	0.047	-	-	-
	Primary male body size	0.011	0.008	1.807	0.178	1
	Secondary male body size	0.012	0.009	1.721	0.191	1
	Female prior treatment [Predator]	0.001	0.009	0.004	0.949	1
	Block [2]	-0.009	0.012	0.689	0.709	2
	Block [3]	-0.008	0.012			
	Female body size at parturition (cm)	0.035	0.006	28.282	**<0.001**	1
	Tank system [2]	0.069	0.010	31.542	**<0.001**	1
	Tank level [Level4]	-0.011	0.016	1.702	0.637	3
	Tank level [Level2]	-0.004	0.012			
	Tank level [Level1]	0.009	0.012			
	Tank centrality [Periphery]	0.010	0.011	0.776	0.378	1
	**Random Effects**					
	σ^2	0.00				
	τ_{00} (Brood ID)	0.00				
	τ_{00} (Primary male ID)	0.00				
	τ_{00} (Secondary male ID)	0.00				
	τ_{00} (Female/Tank ID)	0.00				
	τ_{00} (Female origin)	0.00				
	N (Female/Tank ID)	57				
	N (Primary male ID)	59				
	N (Secondary male ID)	58				
	N (Brood ID)	169				
	N (Female origin)	6				
	Observations	2966				
	Marginal R^2 / Conditional R^2	0.359 / NA				

S4 Table: No link between male size and brood size – when including Phase 1 reproduction. The model structure is identical to the model presented in S2 Table, with the difference that Phase 1 broods were included, where no male overlap during the female's fertile window occurred (i.e., 30 ± 2 days prior to parturition).

<table>
<tr><th>Response</th><th>Predictors</th><th>Estimate</th><th>SE</th><th>χ^2</th><th>p</th><th>df</th></tr>
<tr><td rowspan="23">Brood size</td><td>(Intercept)</td><td>3.810</td><td>10.793</td><td>-</td><td>-</td><td>-</td></tr>
<tr><td>Primary male body size</td><td>0.842</td><td>1.988</td><td>0.179</td><td>0.672</td><td>1</td></tr>
<tr><td>Secondary male body size</td><td>-1.834</td><td>2.138</td><td>0.734</td><td>0.392</td><td>1</td></tr>
<tr><td>Female prior treatment [Predator]</td><td>0.910</td><td>1.572</td><td>0.334</td><td>0.563</td><td>1</td></tr>
<tr><td>Block [2]</td><td>-0.938</td><td>2.072</td><td rowspan="2">0.245</td><td rowspan="2">0.885</td><td rowspan="2">2</td></tr>
<tr><td>Block [3]</td><td>-0.063</td><td>1.860</td></tr>
<tr><td>Female body size at parturition (cm)</td><td>3.797</td><td>1.483</td><td>6.432</td><td>0.011</td><td>1</td></tr>
<tr><td>Tank system [2]</td><td>1.973</td><td>1.705</td><td>1.287</td><td>0.257</td><td>1</td></tr>
<tr><td>Tank level [Level4]</td><td>-2.650</td><td>2.529</td><td rowspan="3">2.893</td><td rowspan="3">0.408</td><td rowspan="3">3</td></tr>
<tr><td>Tank level [Level2]</td><td>-2.745</td><td>2.032</td></tr>
<tr><td>Tank level [Level1]</td><td>-3.082</td><td>2.032</td></tr>
<tr><td>Tank centrality [Periphery]</td><td>-0.247</td><td>1.771</td><td>0.019</td><td>0.889</td><td>1</td></tr>
<tr><td colspan="6">Random Effects</td></tr>
<tr><td>σ^2</td><td colspan="5">97.37</td></tr>
<tr><td>τ_{00} (Primary male ID)</td><td colspan="5">0.00</td></tr>
<tr><td>τ_{00} (Secondary male ID)</td><td colspan="5">0.00</td></tr>
<tr><td>τ_{00} (Female/Tank ID)</td><td colspan="5">0.00</td></tr>
<tr><td>τ_{00} (Female origin)</td><td colspan="5">0.00</td></tr>
<tr><td>N (Female/Tank ID)</td><td colspan="5">57</td></tr>
<tr><td>N (Primary male ID)</td><td colspan="5">59</td></tr>
<tr><td>N (Secondary male ID)</td><td colspan="5">58</td></tr>
<tr><td>N (Female origin)</td><td colspan="5">6</td></tr>
<tr><td>Observations</td><td colspan="5">168</td></tr>
<tr><td></td><td>Marginal R^2 / Conditional R^2</td><td colspan="5">0.071 / NA</td></tr>
</table>
