# Supplementary material for: Paternal effects without paternity? Testing non-genetic male influence on offspring size and brood size in a gynogenetic vertebrate, the Amazon molly (Poecilia formosa)
Source: PLoS One. 2026 Feb 27;21(2):e0328962. doi: 10.1371/journal.pone.0328962 (PMC12948072; doi:10.1371/journal.pone.0328962)
Supplement: S3 File — (PDF) [file pone.0328962.s003.pdf]

Supporting Information 3: Robustness analysis with respect to the exclusion of single-brood males

For:

Paternal effects without paternity? Testing non-genetic male influence on offspring size and brood size in a gynogenetic vertebrate, the Amazon molly (*Poecilia formosa*)

Ulrike Scherer[1,2,3]*, Sean M. Ehlman[1,2,3,4], David Bierbach[1,2,3], Jens Krause[1,2,3] & Max Wolf[1,3]

[1] SCIoI Excellence Cluster, Technische Universität Berlin, Berlin, Germany
[2] Faculty of Life Sciences, Humboldt University, Berlin, Germany
[3] Department of Fish Biology, Fisheries, and Aquaculture, Leibniz Institute of Freshwater Ecology and Inland Fisheries, Berlin, Germany
[4] Department of Biological Sciences, University of South Carolina, Columbia, SC, USA

*Corresponding author: u.k.scherer@gmail.com

S5 Table: Link between primary male size and offspring size – when excluding single-brood males. The model structure is identical to the model presented in S1 Table, except that offspring from broods were excluded, whose assigned primary and/or secondary male did not contribute to any other brood.

Response	***Predictors***	***Estimate***	***SE***	χ^2	***p***	***df***
Offspring size (mm)	(Intercept)	0.511	0.062	-	-	-
	Primary male body size	0.023	0.010	5.161	**0.023**	1
	Secondary male body size	0.012	0.010	1.316	0.251	1
	Female prior treatment [Predator]	-0.006	0.010	0.334	0.563	1
	Block [2]	-0.001	0.018	0.188	0.911	2
	Block [3]	-0.007	0.018			
	Female body size at parturition (cm)	0.029	0.009	9.611	**0.002**	1
	Tank system [2]	0.062	0.011	20.580	**<0.001**	1
	Tank level [Level4]	-0.003	0.016	10.329	**0.016**	3
	Tank level [Level2]	0.004	0.013			
	Tank level [Level1]	0.039	0.013			
	Tank centrality [Periphery]	0.008	0.012	0.333	0.564	1
	**Random Effects**					
	σ^2	0.00				
	τ_{00} (Brood ID)	0.00				
	τ_{00} (Female/Tank ID)	0.00				
	τ_{00} (Secondary male ID)	0.00				
	τ_{00} (Primary male ID)	0.00				
	τ_{00} (Female origin)	0.00				
	N (Female/Tank ID)	47				
	N (Primary male ID)	33				
	N (Secondary male ID)	33				
	N (Brood ID)	90				
	N (Female origin)	6				
	Observations	1733				
	Marginal R^2 / Conditional R^2	0.350 / NA				

S6 Table: No link between male size and brood size – when excluding single-brood males. The model structure is identical to the model presented in S2 Table, except that broods were excluded, whose assigned primary and/or secondary male did not contribute to any other brood.

Response	***Predictors***	***Estimate***	***SE***	χ^2	***p***	***df***
Brood size	(Intercept)	62.761	16.101	-	-	-
	Primary male body size	-2.449	2.565	0.897	0.344	1
	Secondary male body size	-3.029	2.391	1.562	0.211	1
	Female prior treatment [Predator]	1.269	2.027	0.382	0.537	1
	Block [2]	-2.126	2.431	2.962	0.227	2
	Block [3]	-4.208	2.236			
	Female body size at parturition (cm)	-3.527	2.353	2.198	0.138	1
	Tank system [2]	2.787	2.148	1.569	0.210	1
	Tank level [Level4]	-1.945	3.046	12.523	**0.006**	3
	Tank level [Level2]	-4.093	2.540			
	Tank level [Level1]	-9.995	2.421			
	Tank centrality [Periphery]	-1.135	2.216	0.261	0.609	1
	**Random Effects**					
	σ^2	68.62				
	τ_{00} (Female/Tank ID)	0.15				
	τ_{00} (Primary male ID)	7.46				
	τ_{00} (Secondary male ID)	0.00				
	τ_{00} (Female origin)	0.00				
	N (Female/Tank ID)	48				
	N (Primary male ID)	33				
	N (Secondary male ID)	33				
	N (Female origin)	6				
	Observations	90				
	Marginal R^2 / Conditional R^2	0.266 / NA				
