# Supplementary material for: Paternal effects without paternity? Testing non-genetic male influence on offspring size and brood size in a gynogenetic vertebrate, the Amazon molly (Poecilia formosa)
Source: PLoS One. 2026 Feb 27;21(2):e0328962. doi: 10.1371/journal.pone.0328962 (PMC12948072; doi:10.1371/journal.pone.0328962)
Supplement: S4 File — (PDF) [file pone.0328962.s004.pdf]

Supporting Information 4: Robustness analysis with respect to the exclusion of presumably old or poor-condition males

For:

Paternal effects without paternity? Testing non-genetic male influence on offspring size and brood size in a gynogenetic vertebrate, the Amazon molly (*Poecilia formosa*)

Ulrike Scherer[1,2,3]*, Sean M. Ehlman[1,2,3,4], David Bierbach[1,2,3], Jens Krause[1,2,3] & Max Wolf[1,3]

[1] SCIoI Excellence Cluster, Technische Universität Berlin, Berlin, Germany

[2] Faculty of Life Sciences, Humboldt University, Berlin, Germany

[3] Department of Fish Biology, Fisheries, and Aquaculture, Leibniz Institute of Freshwater Ecology and Inland Fisheries, Berlin, Germany

[4] Department of Biological Sciences, University of South Carolina, Columbia, SC, USA

*Corresponding author: u.k.scherer@gmail.com

S7 Table: Link between primary and secondary male size and offspring size – when excluding presumably old or poor-condition males. The model structure is identical to the model presented in S1 Table, except that offspring from broods were excluded, whose assigned primary and/or secondary male died within two months after mating.

Response	***Predictors***	***Estimate***	***SE***	χ^2	***p***	***df***
Offspring size (mm)	(Intercept)	0.383	0.065	-	-	-
	Primary male body size	0.036	0.013	7.531	**0.006**	1
	Secondary male body size	0.009	0.011	0.569	**0.451**	1
	Female prior treatment [Predator]	-0.009	0.010	0.831	0.362	1
	Block [2]	-0.005	0.014	1.507	0.471	2
	Block [3]	-0.015	0.013			
	Female body size at parturition (cm)	0.047	0.010	18.125	**<0.001**	1
	Tank system [2]	0.060	0.011	22.070	**<0.001**	1
	Tank level [Level4]	-0.006	0.015	3.217	0.359	3
	Tank level [Level2]	0.013	0.014			
	Tank level [Level1]	0.021	0.014			
	Tank centrality [Periphery]	0.031	0.012	5.933	**0.015**	1
	**Random Effects**					
	σ^2	0.00				
	τ_{00} (Brood ID)	0.00				
	τ_{00} (Female/Tank ID)	0.00				
	τ_{00} (Secondary male ID)	0.00				
	τ_{00} (Primary male ID)	0.00				
	τ_{00} (Female origin)	0.00				
	N (Female/Tank ID)	43				
	N (Primary male ID)	39				
	N (Secondary male ID)	41				
	N (Brood ID)	82				
	N (Female origin)	6				
	Observations	1596				
	Marginal R^2 / Conditional R^2	0.420 / NA				

S8 Table: No link between male size and brood size – when excluding presumably old or poor-condition males. The model structure is identical to the model presented in S2 Table, except that broods were excluded, whose assigned primary and/or secondary male died within two months after mating.

Response	***Predictors***	***Estimate***	***SE***	χ^2	***p***	***df***
Brood size	(Intercept)	40.475	18.153	-	-	-
	Primary male body size	-0.555	2.789	0.209	0.648	1
	Secondary male body size	-0.169	3.064	1.141	0.286	1
	Female prior treatment [Predator]	1.341	2.212	0.011	0.921	1
	Block [2]	-1.756	2.945	2.582	0.275	2
	Block [3]	-3.646	2.492			
	Female body size at parturition (cm)	-2.563	2.821	0.336	0.562	1
	Tank system [2]	3.123	2.386	3.197	0.074	1
	Tank level [Level4]	-3.152	3.323	7.860	**0.049**	3
	Tank level [Level2]	-6.138	2.979			
	Tank level [Level1]	-10.057	3.127			
	Tank centrality [Periphery]	0.538	2.578	0.068	0.794	1
	**Random Effects**					
	σ^2	85.43				
	τ_{00} (Female/Tank ID)	0.00				
	τ_{00} (Secondary male ID)	0.00				
	τ_{00} (Primary male ID)	0.00				
	τ_{00} (Female origin)	0.00				
	N (Female/Tank ID)	43				
	N (Primary male ID)	38				
	N (Secondary male ID)	41				
	N (Female origin)	6				
	Observations	81				
	Marginal R^2 / Conditional R^2	0.195 / NA				
