# Supplementary material for: Paternal effects without paternity? Testing non-genetic male influence on offspring size and brood size in a gynogenetic vertebrate, the Amazon molly (Poecilia formosa)
Source: PLoS One. 2026 Feb 27;21(2):e0328962. doi: 10.1371/journal.pone.0328962 (PMC12948072; doi:10.1371/journal.pone.0328962)
Supplement: S5 File — (PDF) [file pone.0328962.s005.pdf]

Supporting Information 5: Robustness analysis with respect to selecting most parsimonious models

For:

Paternal effects without paternity? Testing non-genetic male influence on offspring size and brood size in a gynogenetic vertebrate, the Amazon molly (*Poecilia formosa*)

Ulrike Scherer[1,2,3]*, Sean M. Ehlman[1,2,3,4], David Bierbach[1,2,3], Jens Krause[1,2,3] & Max Wolf[1,3]

[1] SCIoI Excellence Cluster, Technische Universität Berlin, Berlin, Germany

[2] Faculty of Life Sciences, Humboldt University, Berlin, Germany

[3] Department of Fish Biology, Fisheries, and Aquaculture, Leibniz Institute of Freshwater Ecology and Inland Fisheries, Berlin, Germany

[4] Department of Biological Sciences, University of South Carolina, Columbia, SC, USA

*Corresponding author: u.k.scherer@gmail.com

S9 Table: The link between primary and secondary male size and offspring size becomes non-significant when selecting the most parsimonious model. Presented are the full model, containing all predictor variables (identical to S1 Table), as well as the most parsimonious model, where non-significant predictor variables were removed in a stepwise backwards model selection procedure.

Response	***Predictors***	**Full model**					**Most parsimonious model**				
		Estimate	***SE***	χ^2	***p***	***df***	***Estimate***	***SE***	χ^2	***p***	***df***
Offspring size (mm)	(Intercept)	0.395	0.055	-	-	-	0.498	0.044	-	-	-
	Primary male body size	0.022	0.009	6.392	**0.012**	1	-	-	-	-	-
	Secondary male body size	0.019	0.009	4.476	**0.034**	1	-	-	-	-	-
	Female prior treatment [Predator]	-0.003	0.010	0.097	0.756	1	-	-	-	-	-
	Block [2]	-0.010	0.012	0.745	0.687	2	-	-	-	-	-
	Block [3]	-0.009	0.012				-	-			
	Female body size at parturition (cm)	0.045	0.008	25.029	**<0.001**	1	0.053	0.008	34.233	**<0.001**	1
	Tank system [2]	0.065	0.011	26.873	**<0.001**	1	0.071	0.010	30.596	**<0.001**	1
	Tank level [Level4]	-0.007	0.015	4.288	0.232	3	-	-	-	-	-
	Tank level [Level2]	0.007	0.013				-	-			
	Tank level [Level1]	0.023	0.013				-	-			
	Tank centrality [Periphery]	0.021	0.012	2.881	0.091	1	-	-	-	-	-
	**Random Effects**										
	σ^2	0.00					0.00				
	τ_{00} (Brood ID)	0.00					0.00				
	τ_{00} (Secondary male ID)	0.00					0.00				
	τ_{00} (Female/Tank ID)	0.00					0.00				
	τ_{00} (Primary male ID)	0.00					0.00				
	τ_{00} (Female origin)	0.00					0.00				
	ICC						0.37				
	N (Female/Tank ID)	53					53				
	N (Primary male ID)	50					50				
	N (Secondary male ID)	56					56				
	N (Brood ID)	127					127				
	N (Female origin)	6					6				
	Observations	2435					2435				
	Marginal R^2 / Conditional R^2	0.393 / NA					0.259 / 0.532				

S10 Table: No link between male size and brood size - when selecting the most parsimonious model. Presented are the full model, containing all predictor variables (identical to S2 Table), as well as the most parsimonious model, where non-significant predictor variables were removed in a stepwise backwards model selection procedure.

Response	***Predictors***	**Full model**					**Most parsimonious model**				
		Estimate	***SE***	χ^2	***p***	***df***	***Estimate***	***SE***	χ^2	***p***	***df***
Brood size	(Intercept)	53.509	13.038	-	-	-	23.881	1.535	-	-	-
	Primary male body size	-1.430	1.977	0.512	0.475	1	-	-	-	-	-
	Secondary male body size	-3.216	2.071	2.366	0.124	1	-	-	-	-	-
	Female prior treatment [Predator]	0.529	1.696	0.097	0.755	1	-	-	-	-	-
	Block [2]	-0.796	2.156	0.883	0.643	2	-	-	-	-	-
	Block [3]	-1.901	2.015				-	-			
	Female body size at parturition (cm)	-2.617	1.971	1.742	0.187	1	-	-	-	-	-
	Tank system [2]	2.489	1.873	1.592	0.207	1	-	-	-	-	-
	Tank level [Level4]	-3.066	2.683	9.102	**0.028**	3	-4.105	2.667	7.969	**0.047**	3
	Tank level [Level2]	-3.375	2.216				-2.909	2.309			
	Tank level [Level1]	-7.189	2.194				-6.941	2.336			
	Tank centrality [Periphery]	-0.550	2.015	0.073	0.787	1	-	-	-	-	-
	**Random Effects**										
	σ^2	72.22					72.98				
	τ_{00} (Secondary male ID)	0.00					0.00				
	τ_{00} (Female/Tank ID)	3.99					7.89				
	τ_{00} (Primary male ID)	1.04					2.52				
	τ_{00} (Female origin)	0.00					0.00				
	N (Female/Tank ID)	53					53				
	N (Primary male ID)	50					50				
	N (Secondary male ID)	56					56				
	N (Female origin)	6					6				
	Observations	126					126				
	Marginal R^2 / Conditional R^2	0.152 / NA					0.089 / NA				
