# Supplementary material for: Paternal effects without paternity? Testing non-genetic male influence on offspring size and brood size in a gynogenetic vertebrate, the Amazon molly (Poecilia formosa)
Source: PLoS One. 2026 Feb 27;21(2):e0328962. doi: 10.1371/journal.pone.0328962 (PMC12948072; doi:10.1371/journal.pone.0328962)
Supplement: Attachment — Submitted filename: Paternal_effects_PLOS_One_revision1_response_letter_2.docx [file pone.0328962.s007.docx]

Dear Dr. Schubert,

We would like to sincerely thank you and the reviewers for the careful and constructive evaluation of our manuscript. The detailed feedback has been invaluable and has led to substantial improvements in the clarity, rigor, and scope of our work.

In our revision, we have comprehensively reworked and expanded the manuscript in response to all reviewer comments. Major changes include:

- A substantially deepened Introduction, now providing detailed background information on potential mechanisms and pathways through which paternal effects may arise in our system, including ejaculate-mediated effects and female differential allocation (lines 53-80).
- A thoroughly revised Methods section, providing clearer and more detailed descriptions of the experimental design and breeding procedures (lines 165-190 and lines 210-236).
- Analytical revisions, where we now account for the potential effects of multiple males (by distinguishing “primary” and “secondary” males), exclude Phase 1 reproduction from the main analyses due to reduced standardization and overlapping male exposures, and control for potential effects of tank position (level and centrality) (lines 269-282).
- Updated Results, reflecting these analytical refinements and presenting several robustness analyses. Notably, we now detect weak, non-genetic male effects, although these effects are small and not robust across all model variants (lines 305-402).
- A rewritten Discussion, where we substantially toned down our conclusions, emphasizing the tentative nature of our findings, and acknowledging key limitations like the use of a single clonal lineage, the limited generality of our results, and the absence of experimental manipulations of male experience (lines 405-474).

Furthermore, in our Acknowledgement section, we have stated the role the funders took in the study: „The funders had no role in study design, data collection and analysis, decision to publish, or preparation of the manuscript."

We believe that these revisions have significantly strengthened the manuscript and that we have addressed all concerns raised by the reviewers. We have included (i) a detailed response to reviewers, (ii) a tracked-changes version of the manuscript, and (iii) a clean version of our manuscript.

We thank you again for the opportunity to revise our work and we hope that the revised manuscript now meets the journal’s and reviewers’ expectations.

With kind regards,

Ulrike Scherer

(on behalf of all co-authors)

# Reviewer #1:

This manuscript presents a study examining paternal effects in the gynogenetic Amazon molly. The research question is intriguing and relevant, and the manuscript is generally well written. However, I have several concerns regarding both methodology and analysis that need to be addressed to strengthen the validity of the findings. The core issue lies in the interpretation of paternity in a system where males do not contribute genetically, raising questions about the assignment of paternal identity. Additionally, there are analytical and reporting elements that require clarification or revision.

\# We sincerely thank the reviewer for their thoughtful and constructive feedback. Their careful evaluation and insightful suggestions have substantially strengthened the clarity, precision, and overall quality of our manuscript. We greatly appreciate the time and expertise they invested in this review.

Major Comments:
1. Interpretation of “Paternity” in Gynogenetic Species:
It is problematic to frame the analysis around the assignment (or lack thereof) of paternity to specific males when these males do not contribute genetic material. I recognise that this is probably a simpler way to frame it, to call it sired, fathered, etc. rather than calling it the identify of males that were housed with females, but it’s not technically accurate? Also, as females were exposed to multiple males across time, stored sperm may influence brood outcomes, meaning the most recent male may not be the one affecting brood traits. While the manuscript cites evidence for strong fertilization bias, this comes from a different species (P. reticulata), which is not gynogenetic. It is unclear whether sperm precedence operates similarly in the Amazon molly. The authors should clarify this assumption or provide more robust justification for its applicability here, and address this potential limitation in the discussion.

\# Regarding the first point raised (terminology around paternity): We thank the reviewer for this important comment and for drawing attention to the nuanced interpretation of “paternity” in gynogenetic systems. We fully agree that terms such as “sired” or “fathered” can be misleading in a system where males do not contribute genetically to reproduction. To avoid this ambiguity, we are now explicitely acknowledge the paradox of testing for paternal effects in a species without genetic paternity – both in the revised title of our manuscript (lines 2-3) as well as in the Introduction section (lines 92-94). Furthermore, we have now revised the manuscript to phrase this aspect more carefully. Specifically, we are now referring to ‘males that have been assigned to offspring/broods’. For example:

Original manuscript (lines 154-155): “*For each brood, the father was identified as the male who was in the female’s tank 30 days before parturition.*”

Revised manuscript (lines 211-214): “*For each brood, the male who was in the female’s tank 30 days before parturition was identified as the ‘primary male’ […] this male most likely triggered embryonic development of the brood in question*.”

Original manuscript (lines 224-226): “*However, our results are robust with respect to the removal of offspring or broods, which were sired by males who died within two months post pseudo-fertilization…*”.

Revised manuscript (lines 376-377): *“…we repeated our main analysis excluding all broods and offspring assigned to males (primary and secondary) that died within two months after mating.*”.

Regarding the second point raised (sperm storage): We thank the reviewer for highlighting this important point. We fully agree that our original manuscript did not sufficiently consider the potential role of multiple males’ sperm being present in the female reproductive tract at the time of insemination or during embryonic development. In our revised version, we have now explicitly addressed this issue by identifying not only the male most likely to have triggered embryonic development based on the timing of exposure (the “primary male”) - but also the male whose sperm was second most likely to have influenced embryonic development (“secondary male”) (lines 210-221). We have not identified third and fourth males because our sample size is limited and because we expect more distant encounters with males to be of less importance (lines 219-221). In our statistical analyses, we now test for potential effects of both the primary and secondary males’ identities or body sizes (lines 279-280). In short, we now find significant, yet weak, effects of both males (primary and second) (lines 306-315).

2. Tank Effects:
The manuscript mentions a test for “tank system” but does not appear to include individual tank identity (tank ID) as a factor. This is potentially problematic, as tank-level variation could confound the results. A random effect for tank ID should be included, or at minimum, tank effects should be explicitly tested and reported.

\# We thank the reviewer for this important comment and fully agree that tank-level variation can represent a potential confounding factor. In our experiment, each female was housed in a single, uniquely assigned tank throughout the entire study, meaning that female identity and tank identity were synonymous. Accordingly, our statistical models already included this factor as a random effect, called “female identity”. In the revised manuscript, we have now clarified this point in the Methods section of the revised manuscript (lines 151-155 and lines 279-281) and replaced “female identity” with “female/tank identity” wherever applicable (lines 280 and 284, Supporting Information S01 – S10 Table).

In addition, following the reviewer’s suggestion, we have now extended our statistical models to account for potential systematic spatial variation among tanks. Specifically, while we have controlled for variation among tank systems in our original manuscript, we are now also controlling for tank level (tanks were distributed over four vertical levels) and tank centrality (tanks were classified as central, with neighboring tanks on both sides, or peripheral, i.e., edge tanks with only one neighbor) as fixed covariates in all analyses (lines 272-276, Supporting Information S01 – S10 Table). This expanded model structure ensures that any systematic environmental differences across tank positions are appropriately controlled for.

3. Inclusion of Males with Only One Brood:
Figure 1 indicates that some males sired only a single brood. What is the justification for including these males in the repeatability analysis? These single observations may not contribute meaningfully to the estimation of variance components and could dilute the signal. The authors should test whether excluding these cases changes the results and report accordingly. Likewise, there are a lot of factors in the models, many of which are not significant. Have you tried running he models without non-significant fixed effect/covariates? Though I note that it doesn’t seem like it will change the results based on the data plotted in the figures.

\# Regarding the first point raised (the inclusion of males with only one brood): All males were included in the repeatability analysis, including those who produced only a single brood, to ensure that the full distribution of reproductive outcomes in the population was accurately represented. To address the reviewer’s concern, we have now clarified this rationale in the Results section and additionally conducted a robustness analysis excluding single-brood males. In short, our results were largely robust to the exclusion of these males, except that the primary male’s repeatability for brood size increased notably (from roughly 2% to 12%; lines 355-371, Supporting Information 3). In our revised manuscript, we clearly highlight the difference in results between our main analysis including all males vs. the analysis without single-brood males and discuss its implications (lines 355-371, lines 418-426).

Regarding the second point raised (removal of non-significant predictors): We have now tested the robustness of our results with respect to the removal of non-significant factors using backward model selection. Our results were largely robust to the exclusion of non-significant fixed effects, except that the weak association between male body size and offspring size reported in our main analyses became non-significant (lines 385-392). In the revised manuscript, we now clearly highlight the differences among these analytical variants and discuss their implications (lines 418-426).

4. Random Effects Structure:
It is unclear why the interaction between male and female identity was not included as a random effect. Given the experimental design, such an interaction could capture potential dyad-specific variation in brood traits?

\# We thank the reviewer for this thoughtful suggestion. We agree that, in principle, including a male×female interaction term could capture variation that is specific to a particular pairing. However, in our experimental design, each male was paired with a female only once, resulting in a single brood per unique male–female combination. As there are no repeated measurements within these combinations, the male×female interaction cannot be estimated as a random effect in a meaningful way. For this reason, our models include male ID and female ID as random effects, but not their interaction. In the revised manuscript, we have now added this explanation to the description of model structures (lines 286-289).

5. Experimental Design: It would help if you could state how often females received a new unfamiliar male - it was every two weeks, but for how long, continuously from 70 to 260 days old for some females? And this was just until they produced the 1st brood, yes, and then it was a new male after every new brood? Or did the 2-week cycle start again for this next brood? On line 138 it says that in phase 2, an unfamiliar male was added whenever she gave birth to a new brood, but this was 7 days after giving birth to the new brood, yes?

\# We thank the reviewer for this helpful comment, which prompted us to clarify our description of the experimental procedure. In the revised Methods section, we now provide a more detailed explanation of how males were introduced during the two phases of the breeding design and why two different breeding procedures were used (lines 165-189). Specifically, we explain that in Phase 1, beginning at an age of 70 days, each female was continuously paired with an unfamiliar male, who was replaced every two weeks until she produced her first brood. After the first brood, the current male remained for an additional seven days to provide fresh sperm. In Phase 2, we then introduced a new unfamiliar male only when a female gave birth to a new brood, leaving the male in the tank for exactly seven days. While Phase 1 was necessary to effectively initiate reproduction, the transition to Phase 2 minimized potential confounds arising from sperm of multiple males being simultaneously present within a female’s reproductive tract.

To further enhance clarity, we have added a schematic of our experimental procedure (Figure 1) that visually depicts the experimental procedure across both phases, and we now also specify the duration of phase 1 by reporting the mean age at first reproduction for females in the final dataset (*N* = 54 females, 181.1 ± 39.8 days; lines 175-176).

We hope that these changes make the design and timing of male introductions fully transparent.

Minor Comments / Line-by-Line Suggestions:

• Line 54: Add references to support the statement: “…from insects to mammals.”

\# In the revised manuscript, we have now added references for this statement (line50).

• Line 70: Clarify wording. The cited study did not find significant effects of male species; it reported a non-significant trend in the allopatric vs. sympatric comparison.

\# In the revised manuscript, we have now rephrases this sentence accordingly (lines 96-100).

• Line 93: Replace “mollies” with “molly.”

\# Corrected (line 143).

• Line 118: Replace “juveniles” with “juvenile.”

\# Corrected (line 151).

• Line 133-138: Clarify the male exposure schedule. Was an unfamiliar male introduced every two weeks from 70 to 260 days of age, or just until the first brood? After the first brood, were females exposed to a new male each time they reproduced? Line 138 states that the new male was introduced seven days after parturition—this needs to be clearer and more consistent with earlier descriptions.

\# In the revised Methods, we now clearly describe how males were introduced in each phase - continuous two-week rotations until first reproduction in Phase 1, followed by seven-day introductions after each brood in Phase 2 (lines 165-182). To further aid clarity, we have added a schematic of the experimental procedure (Figure 1).

For a more detailed answer see also our response to Reviewer 1’s above comment #5.

• Line 148: Remove “a” from “the day they gave a birth.”

\# Corrected (line 193).

• Line 178: The sentence “no effect of either factor” is followed by three factors. Please revise for consistency.

\# In the revised manuscript, we have now rephrased to “none of these factors significantly affected…” (line 259).

• Line 191: Remove the comma: change “(32), (33)” to “(32,33).”

\# Corrected (line 265).

• Line 197: Add “the” before “response.”

\# Corrected (line 271).

• Line 211-212: Why is average male length used? If brood-specific male length was measured, it would seem more appropriate and accurate to use that value.

\# We used average male length rather than brood-specific male length, as poeciliid males cease, or at least markedly reduce, somatic growth upon reaching sexual maturity. Variation in repeated size measurements of the same individual is therefore more likely to reflect measurement error than true growth. Consequently, we considered average length the more robust measure. We have now included this explanation in the revised manuscript (line 298-302). Furthermore, we included a supplementary figure depicting all individual size measurements (S1 Fig).

• Line 217: Clarify that repeatability estimates of zero reflect zero variance explained by male identity in the models.

\# In the revised manuscript, we now explain that repeatability represents the proportion variation explained by male identity (line 291-293 and lines 306-307).

• Line 225: Explain the rationale for excluding males that died within two months. Was this to account for potential illness affecting reproductive interactions?

\# We have conducted this robustness analysis to account for potential effects of age or general body condition. In the revised manuscript, we now added this explanation (line 375-377).

Figures and Supplementary Material:

• Figure 1: Please clarify why the figure shows a single data point per brood. Would it not be more informative to show average brood size or trait value, especially if multiple offspring were measured?

\# We thank the reviewer for this helpful comment. We understand this suggestion as proposing to display average trait values per individual male (i.e., one data point per male). While we agree that such an approach would clearly emphasize among-male differences in mean trait values, our goal with this figure was to illustrate the raw data structure underlying our analyses, including both within-male and within-brood variation. Plotting only male-level means would obscure this brood-level structure and the natural variation among broods that is central to our analysis. To better represent these hierarchical patterns, we have retained the brood-level data but now additionally display error bars for offspring sizes indicating within-brood standard deviations, thereby providing maximum transparency regarding the observed variability in our data (now Fig 2).

• Supplementary Material: Consider revising the layout of the random effects and associated sample sizes. Instead of using rows to indicate sample sizes, include the sample size alongside each random effect name for clarity.

\# We thank the reviewer for their thoughtful suggestion regarding the presentation of the random effects and associated sample sizes. We agree that clarity in supplementary tables is important. In this manuscript, however, we feel that the current format, where sample sizes and variances are stated separately, already communicates the relevant information clearly, and we would therefore prefer to retain it as is. We very much appreciate the idea, though, and will certainly keep it in mind for future manuscripts.

# Reviewer #2:

This paper looks at the effects of paternal identity on offspring traits in a gynogenetic system, where males do not contribute genetically to the production of offspring. The authors found that male identity did not significantly predict offspring size or brood size. While the writing was clear and concise, I have a number of significant issues with the experimental design and manuscript.

\# We sincerely thank the reviewer for their thoughtful and constructive feedback. Their careful evaluation and insightful suggestions have substantially strengthened the clarity, precision, and overall quality of our manuscript. We greatly appreciate the time and expertise they invested in this review.

1. We generally need more background about the mechanisms by which sperm can induce paternal effects, both when the egg is fertilized and when it is not. For example, what are typical mechanisms that fathers use to influence offspring and which ones would be potential mechanisms that could still occur even if the father did not contribute genetic material? Some of this background needs to be species specific. What happens to the sperm in this system- does it actually fertilize the egg (such that non-coding RNAs could influence gene expression in the developing embryo) or is the sperm just in the vicinity (such that proteins in seminal fluid might be important)?

\# We thank the reviewer for this important comment and for prompting us to expand the mechanistic background on how paternal effects can arise in our system - both with and without fertilization. In our substantially revised Introduction section, we now provide a clearer overview of ejaculate-mediated pathways, distinguishing between sperm-borne epigenetic mechanisms and seminal fluid–mediated mechanisms (lines 53-80).

We have also clarified what happens to sperm in the Amazon molly system. Specifically, sperm does fuse with the oocyte to trigger embryogenesis, but the male’s genetic material is subsequently excluded from the developing embryo (lines 88-90). This makes both sperm-borne and seminal-fluid–mediated effects plausible routes for paternal influence despite the absence of genetic paternity.

2. I found the literature review and introduction to generally be a bit lacking in detail. There are a number of studies in the sperm competition literature about the role of a secondary male’s sperm in the vaginal tract and how it affects their non-biological offspring. This would be important to draw on. Including some of this information would also give a better background about why this is important. On one hand, this is a unique system for looking at these effects, but on the other hand, it is possible that these effects are not generalizable beyond this species.

\# We thank the reviewer for highlighting this important point. We fully agree that our original manuscript did not sufficiently consider the potential role of multiple males’ sperm being present in the female reproductive tract at the time of insemination or during embryonic development. In the revised version, we have addressed this in two ways. First, in the Introduction section, we now highlight how seminal fluid from multiple males can affect offspring traits and fertilisation success - even when genetic paternity is excluded (lines 53-74). Second, analytically, we now explicitly account for the influence of multiple males by identifying both the male most likely to have triggered embryogenesis based on the timing of exposure (the “primary male”) and the male whose sperm was second most likely to have contributed (“secondary male”) (lines 210-222). In our statistical analyses, we now test for potential effects of both the primary and secondary males’ identities or body sizes (lines 279-281). In short, we now find significant, yet weak, effects of both males (primary and second) (lines 306-315). We have not identified third and fourth males because our sample size is limited and because we expect more distant encounters with males to be of less importance (lines 219-221).

Additionally, we now clearly acknowledge the fact that our results may not be generalizable beyond this species by explicitly by adding a note on that regard to our Discussion section (lines 454-464).

3. Why those traits? Why would male identity affect offspring size or brood size? You partially address this, but those seem to be traits that would be much more likely to be linked to female body condition or size. Brood size is largely determined by female size, and then individual offspring size is a reflection of brood size. Indeed, if we looked at paternal effects in many species, we probably would see no effect of paternal identity on these traits (I don’t, in my system), yet we still see very strong effects of paternal experiences.

\# We thank the reviewer for this thoughtful comment. We fully agree that major factors shaping offspring and brood size are maternal condition and investment. However, these traits may also be susceptible to paternal influences through ejaculate-mediated mechanisms that affect fertilization success or embryonic development; or through differential female allocation in response to male phenotype. In the revised manuscript, we now clarify this rationale to justify our choice of traits (lines 110-112). We also acknowledge that other offspring traits - such as physiology or behavior - may be more sensitive to paternal influences and highlight these as promising directions for future research (lines 445-452 and line 472).

4. The methods are very unclear about the relationship of the females to each other. It seems like the females are all genetically identical to each other, rather than coming from separate lineages. Therefore, there is an issue with pseudoreplication- you can conclude that paternal effects do not matter (for the traits you measured) only in this one particular lineage, but it could be that this one particular lineage or population does not respond to males, but others do.

\# The reviewer is right. All females in our experiment originating from a single clonal lineage. We have now clarified this explicitly in the Methods section (lines 252-253). Specifically, in the original manuscript, we stated that all experimental females originated from *N* = 6 mothers, which were themselves full siblings derived from a single brood (lines). To further clarify, in the revised manuscript, we have now added a statement noting that the females therefore represent a single lineage (lines 250-252).

We fully agree that this limits the generality of our conclusions to one lineage. In the revised manuscript, we now clearly acknowledge this limitation and emphasize that our findings apply specifically to this clone, while additional work using multiple clonal lineages will be essential to determine whether paternal effects vary across genotypes or populations (lines 454-464).

5. Generally, the result feel overstated on Line 279-291. While in many ways, the experimental design is neat, the authors have only ruled out the effects of male ID on two traits at birth, but not on traits later in life (which you state). Importantly, the authors didn’t look for the effects of paternal experiences by manipulating a stressor -just looked for the effects of male ID (which are different types of paternal effects). Given that this was tested in only one lineage, the results need to be much more cautious and the title of the paper should reflect that.

\# We thank the reviewer for this thoughtful comment and fully agree that our conclusions should be interpreted with appropriate caution. In the revised manuscript, we have now substantially tempered the tone of our Discussion section to more accurately reflect the scope of our findings (lines 406-474). We now clearly present the lack of experiential differences among males as a major limiting factor of our study (lines 428-437) and acknowledge the importance to look at further offspring phenotypic traits (lines 445-452). In line with this, we have revised the title to more accurately capture the scope and focus of our study: “Paternal effects without paternity? Testing non-genetic male influence on offspring size and brood size in a gynogenetic vertebrate, the Amazon molly (*Poecilia formosa*)” (lines 2-3).

Minor comments
Line 20- I would consider this definition to be pretty far removed from the papers that are cited. It is unclear what is meant by the integration of genetic material- I would just say that that they are not related to the parental genotype.

\# We have now rephrased our definition of paternal effects in the abstract (lines 19-20) as well as in the introduction section (lines 42-43) of our revised manuscript. The definition we now use follows the one provided in Crean & Bonduriansky (TREE, 2014) and Rutkowska et al. (BMC Biology, 2020) (reference number 1 and 3 in the revised manuscript).

Lines 133-143; it is unclear why there are phases to reproduction, this should be justified. In phase 1, did you get rid of the babies? Also, if part of the paternal effects could be triggered by behavioral interactions with the male, your experimental design, with each female interacting with multiple males, probably couldn’t fully detect this because investment in offspring could be the result of her interaction with the previous male, not the current male.

\# We thank the reviewer for this important comment. We fully agree that conditions during Phase 1 were less standardized, particularly due to frequent male rotations and overlapping exposures during females’ receptive periods. This experimental structure is indeed expected to reduce our ability to reliably detect paternal effects. In the revised manuscript, we now justify the rationale behind the two breeding phases and explicitly acknowledge the reduced interpretability of Phase 1 data (lines 179-182 and lines 343-346). Consequently, we decided to exclude Phase 1 reproduction from our final data set. However, we retain a robustness analysis that includes Phase 1 broods. When excluding Phase 1 data, we observe a weak, yet significant, link between male size (primary and secondary) and offspring size (lines 306-315). However, when including Phase 1 data, that link becomes non-significant (lines 343-353). In our revised manuscript, we clearly highlight the difference in results between our main analysis excluding Phase 1 vs. the analysis including Phase 1 and discuss its implications (lines 418-426).

Line 137- what is pseudo-fertilization?

\# Pseudo-fertilisation refers to a process where sperm activates the egg and triggers embryonic development without actually contributing genetic material to the offspring. In the revised manuscript, we have now rephrased in order to avoid obscurities (lines 174-175).

Line 153- need a citation or details about the custom software.

\# In the revised manuscript, we have now added a citation (lines 199-200).

Lines 217-219- need statistical test for this repeatability and more details about how you set this up

\# In the revised manuscript, we have now explained that repeatability reflects the proportion of variation explained by differences among males (relative to the total variation in the population (lines 289-293). We have further added a statement, explaining how significance is derived: repeatability is significant when the 95% confidence intervals do not overlap with zero (lines 293-294).
