# Supplementary material for: Paternal effects without paternity? Testing non-genetic male influence on offspring size and brood size in a gynogenetic vertebrate, the Amazon molly (Poecilia formosa)
Source: PLoS One. 2026 Feb 27;21(2):e0328962. doi: 10.1371/journal.pone.0328962 (PMC12948072; doi:10.1371/journal.pone.0328962)
Supplement: Attachment — Submitted filename: response_letter.docx [file pone.0328962.s008.docx]

**Reviewer #1**

Excellent work revising the manuscript. I have just a few comments for the authors to consider.

\# We thank the reviewer for their positive evaluation of our revision.

Line 34: Just say “this effect was minimal”. Same as at line 311.
\# Corrected (now lines 34 and line 427).

Line 125: What does it mean to be second-most likely to be involved? Involved how?
\# We agree that the original phrasing was insuffiently precise. In the revised manuscript, we have rephrased the sentence to clarify that “second-most likely to be involved” refers specifically to the likelihood that a male’s sperm triggered embryogenesis (now line 125).

Line 140 and line 213: I would actually argue here that you’re less like to see this sperm precedence in the molly though. The males do not contribute genetically to fertilisation, so there’s been no selective pressure for post-copulatory sperm competition – presumably they would just be a mix in the reproductive tract. On the other hand, I would think there would have been strong selection for sperm longevity within the reproductive tract, to ensure that females have sperm available if they don’t encounter a male.

\# We thank the reviewer for this thoughtful comment. We agree that, in a gynogenetic system, there is no direct selective pressure on males for post-copulatory sperm competition in the strict sense, given that male genetic material is not incorporated into the offspring. However, we note that Atlantic molly males used in our study evolve primarily within a sexually reproducing system, in which post-copulatory processes are subject to selection. As a result, sperm traits shaped by sperm competition in the sexual context may still be expressed when these males mate with Amazon molly females, even if they do not contribute genetically to reproduction in this particular interaction. At the same time, we agree that female-mediated post-copulatory processes may be dampened in the Amazon molly compared to sexually reproducing species. We have therefore revised the manuscript to explicitly note that it remains unclear whether such fresh-sperm precedence operates to the same extent in the Amazon molly (now lines 141-142).

We further agree with the reviewer that selection for sperm longevity is likely to be important in this system, ensuring that females have access to sperm even in the absence of male encounters. We believe that sperm longevity is already accounted for in our analyses through the explicit consideration of both primary and secondary males.

Line 175: I like the new figure 1, though arguably it could just be in the supplement. I’m trying to full understand the text and figure. The females produced a brood, the male stayed with the female for seven more days. The female was then left alone until she produced a second brood, and then the male was added? So in the figure, male 3 triggered the 2nd brood. So male 4 is the trigger for brood 3. This is confirmed at line 215. So the analyses are all based on broods 2+. If that is not correct, then I’m still missing something. Why is there a brood #1 plotted in figure 2? On that note, why brood number on the x-axis – why not male identity?
\# We agree with the reviewer that the new Fig. 1 could be a supplementary figure. However, we believe that understanding the experimental procedure - particularly the definition of primary and secondary males - is essential for understanding the results, and this information is most accessible when presented visually (as not all readers will read the detailed Methods section). Therefore, keeping it in the main text adds substantial value.

The reviewer’s interpretation of the breeding protocol is entirely correct: after the first brood, the male remained with the female for seven additional days, after which the female was housed alone until the next brood, at which point a new male was introduced; and our main analyses are indeed based exclusively on brood numbers ≥2. To make this clearer, we have revised both the Methods section (lines 169-171, 176-183) and Fig. 1 description (lines 192-196). In addition, we have updated Fig. 1 to visually emphasize that the first brood is not included in the main analyses.

Regarding the illustration of Brood 1, we agree that without explicit clarification this is misleading, as Phase 1 reproduction is excluded from the main analyses. To address this, we have now highlighted Brood 1 in a different color and added a note to the figure caption, stating that this brood is shown for completeness only and is not included in our main analyses (lines 195-196).

Finally, with respect to the x-axis in Fig. 2, we agree that an alternative visualization using male identity on the x-axis would be possible. However, we believe that the current representation conveys the repeated-measures structure of the data and the underlying experimental logic equally well.

Line 222: Why would there not be a male in the tank 30 days prior?
\# In these cases, no male was present in the female’s tank 30 days prior to parturition because we did not record a brood at that time. We have revised the sentence to clarity this point (lines 230-231).

Line 270: Do you mean 128 as noted above at line 234?
\# We thank the reviewer for catching this inconsistency. The correct sample size is 127 broods (not 128, as stated in the previous line 234). In the revised manuscript, we have corrected this (now line 243).

Line 391: Check p-value, 0.509? Is it 0.0509?
\# We thank the reviewer for carefully checking this value. The reported p-value of 0.509 is correct and not a typo. We detected a trend for the secondary male only. The original wording was misleading in this regard. We have now revised the sentence to accordingly (now line 403-404).

Figure 2: Given that these are different males – should there be lines joining the points?
\# In Fig. 2, points corresponding to broods from the same male are indeed connected by lines to indicate repeated contributions by the same male across broods. We suspect that this may not have been clearly visible in the previously uploaded version of the figure, potentially due to file resolution or formatting issues during submission. We have therefore uploaded a new, higher-quality version of Figure 2 and hope that this resolves the issue and makes the connecting lines clearly visible.

In your figures, it might be worth defining primary and secondary males, so that people don't need to read the methods in detail. Something simple like, male the female was housed with and previous male the female was housed with? Something like that?

\# We thank the reviewer for this helpful suggestion. We agree that defining primary and secondary males directly in the figure caption improves clarity. We have now added such a brief explanatory note to the figure caption (lines 337-340, 350-354).

We would like to thank the reviewer once again for their thoughtful and constructive feedback. Their careful reading and insightful comments were instrumental in improving the overall quality of the manuscript, and we are very grateful for the time and effort they invested in this review.

# Reviewer #2

Overall, I found the paper to be much improved and applaud the authors on their careful revisions. I have a few, very minor comments, but am happy with the revisions!
\# We thank the reviewer for their positive evaluation of our revision.

Lines 27-28- I would remove the sample sizes from the abstract, because it feels misleading given that they all descended from one female
\# We agree that clarity about the biological origin of the study animals is essential, and we have therefore now added the information that all Amazon molly females derive from a single clonal lineage to the abstract (line 28). We would, however, prefer to retain the sample sizes in the abstract. The numbers are intended to convey the scale and experimental effort of the breeding design - particularly the large number of broods and offspring generated and the extensive involvement of different males - rather than to imply independent female genetic backgrounds. We therefore feel that, with the added clarification, the sample sizes remain informative and appropriate to include in the abstract.

Line 79- red, not read
\# Corrected (now line 79).

Line 110- expected
\# Corrected (now line 110).

Line 381- remove the ‘and’. Generally, there are a number of sentences that start with ‘and’ that are incomplete sentences.

\# In the revised manuscript, we have corrected the incomplete sentence in line 381 (now line 394). Additionally, we have corrected all other incidences with the same pattern (now lines 78, 426, 461).

We would like to thank the reviewer once again for their thoughtful and constructive feedback. Their careful reading and insightful comments were instrumental in improving the overall quality of the manuscript, and we are very grateful for the time and effort they invested in this review.
